# The XBB.1.5 slightly increase the binding affinity for host receptor ACE2 and exhibit strongest immune escaping features: molecular modeling and free energy calculation

**DOI:** 10.3389/fmolb.2023.1153046

**Published:** 2023-05-31

**Authors:** Muhammad Suleman, Aneela Murtaza, Maria, Haji Khan, Farooq Rashid, Abdulrahman Alshammari, Liaqat Ali, Abbas Khan, Dong-Qing Wei

**Affiliations:** ^1^ Center for Biotechnology and Microbiology, University of Swat, Swat, Pakistan; ^2^ Nawaz Sharif Medical College, Gujrat, Punjab, Pakistan; ^3^ D. G. Khan Medical College, D. G. Khan, Punjab, Pakistan; ^4^ Division of Infectious Diseases, Chongqing Public Health Medical Center, Chongqing, China; ^5^ Department of Pharmacology and Toxicology, College of Pharmacy, King Saud University, Riyadh, Saudi Arabia; ^6^ Fisch College of Pharmacy, The University of Texas at Tyler, Tyler, TX, United States; ^7^ Department of Bioinformatics and Biostatistics, School of Life Sciences and Biotechnology, Shanghai Jiao Tong University, Shanghai, China; ^8^ Zhongjing Research and Industrialization Institute of Chinese Medicine, Zhongguancun Scientific Park, Meixi, Nayang, Henan, China; ^9^ Peng Cheng Laboratory, Shenzhen, Guangdong, China

**Keywords:** SARS-CoV-2, XBB.1.5 variant, ACE2, antibody, free energy, immune evasion

## Abstract

**Introduction:** The current XBB variant of SARS-CoV-2 with the strongest immune escaping properties is currently the most dominant variant circulating around the world. With the emergence of XBB global morbidities and mortalities have raised again. In the current scenario, it was highly required to delineate the binding capabilities of NTD of XBB subvariant towards human neutralizing antibodies and to dig out the binding affinity of RBD of XBB subvariant with ACE2 receptor.

**Materials and Methods:** The current study uses molecular interaction and simulation-based approaches to decipher the binding mechanism of RBD with ACE2 and mAb interaction with NTD of the spike protein.

**Results:** Molecular docking of the Wild type NTD with mAb revealed a docking score of −113.2 ± 0.7 kcal/mol while XBB NTD docking with mAb reported −76.2 ± 2.3 kcal/mol. On the other hand, wild-type RBD and XBB RBD with ACE2 receptor demonstrated docking scores of −115.0 ± 1.5 kcal/mol and −120.8 ± 3.4 kcal/mol respectively. Moreover, the interaction network analysis also revealed significant variations in the number of hydrogen bonds, salt-bridges, and non-bonded contacts. These findings were further validated by computing the dissociation constant (KD). Molecular simulation analysis such as RMSD, RMSF, Rg and hydrogen bonding analysis revealed variation in the dynamics features of the RBD and NTD complexes due to the acquired mutations. Furthermore, the total binding energy for the wild-type RBD in complex with ACE2 reported −50.10 kcal/mol while XBB-RBD coupled with ACE2 reported −52.66 kcal/mol respectively. This shows though the binding of XBB is slightly increased but due to the variation in the bonding network and other factors makes the XBB variant to enter into the host cell efficiently than the wild type. On the other hand, the total binding free energy for the wildtype NTD-mAb was calculated to be −65.94 kcal/mol while for XBB NTD-mAb was reported to be −35.06 kcal/mol respectively. The significant difference in the total binding energy factors explains that the XBB variant possess stronger immune evasion properties than the others variants and wild type.

**Conclusions:** The current study provides structural features for the XBB variant binding and immune evasion which can be used to design novel therapeutics.

## Introduction

Severe acute respiratory syndrome coronavirus 2 (SARS-CoV-2) emerged in late 2019 and is the cause of the pandemic of coronavirus disease 2019 (COVID-19) (Hu et al., 2022). It is highly transmissible and pathogenic, and since its first emergence several variants have emerged due to the coinfection of an individual with different variants (Shrestha et al., 2022). So far, five variants of concern (VOCs), i.e., Alpha, Beta, Gamma, Delta, and Omicron have been reported so far, however, among all these five variants, Omicron is the most divergent from the Wuhan-Hu-1. Omicron (B.1.1.529 or BA.1) was designated as a variant of concern (VOC) by the world health organization (WHO) on 26 November 2021 (Callaway, 2022) (Callaway and Ledford, 2021) (Cao et al., 2022). The BA.1 and its derivative became dominant globally after its first detection on 21 November 2021, in South Africa (Viana et al., 2022) (Wang et al., 2022a). After the wave of BA.1, subvariant BA.2 became dominated worldwide, and since then BA.4, BA.5, and BA.2.12.1 were detected in different parts of the world (Tegally et al., 2022) (Wang et al., 2022b). XBB is a result of recombination between two lineages of BA.2, i.e., BJ.1 and BA.2.75, which was first identified in India in August 2022 and subsequently spread to Singapore and other Asian countries (Wang et al., 2022b). All the Omicron sub-variants share various mutations and have unique mutations as well (Viana et al., 2022). The XBB has 14 more mutations in addition to the mutations found in BA.2 in its spike protein. Among these new 14 mutations, 5 were in the N-terminal domain (NTD) and 9 in the receptor binding domain (RBD) (Wang et al., 2022b). The NTD region is involved in the binding of human-neutralizing antibodies while the RBD region of the spike protein is involved in the binding with the human angiotensin-converting enzyme 2 (ACE2) receptor. The huge burden of mutations in spike protein has raised serious concerns about the antigenic properties and the efficacy of currently used vaccines against SARS-CoV-2. Neutralizing antibodies against SARS-CoV-2 infection are strongly predictive of the degree of immune protection (Khoury et al., 2021). Previous studies have described that BA.1, BA.2 and BA.3 sub-variants of Omicron show significant resistance to neutralizing antibodies triggered by natural infection, vaccination, and therapeutic monoclonal antibodies (Arora et al., 2022) (Cao et al., 2022) (Carreño et al., 2022) (Evans et al., 2022) (Planas et al., 2022).

In the current scenario, it was highly required to delineate the binding capabilities of NTD of XBB sub-variant towards human neutralizing antibodies and to dig out the binding affinity of RBD of XBB sub-variant with ACE2 receptor by comparing to wild-type NTD and RBD, respectively. Therefore, we used molecular docking and simulation approaches to determine structural determinants that help in escaping the neutralizing antibodies by NTD and improving the binding affinity of RBD towards ACE2 by XBB sub-variant. Interestingly we found that indeed the XBB sub-variant of Omicron is immune escaping and showed stronger binding capabilities towards ACE2 compared to their wild-type counterparts.

## Materials and methods

### Structures retrieval

The newly emerged XBB sub-variant of SARS-CoV-2 is reported to be more contagious and has immune evasion characteristics due to novel mutations in the N-terminal domain and receptor binding domain of spike protein. Concerns have been raised that the quick emergence of these sub-variants and their wide variety of spike mutations may further jeopardize the effectiveness of existing COVID-19 vaccines and monoclonal antibody therapies (Wang et al., 2022b). Therefore, to dig out the immune evasion and higher infectivity of the XBB sub-variant, we retrieved the wild-type complex (ID: 7C2L) of the N-terminal domain (NTD) and human monoclonal antibody (4A8) from the UniProt database (https://www.uniprot.org/) (Magrane, 2011). Afterward, to further check the effect of the XBB mutation on the binding of spike protein to human ace2 receptor, we retrieved the sequence of RBD (319–541aa) from the wild-type spike protein (P0DTC2) available in the UniProt database (Magrane, 2011). Then the modeler 14.0 embedded in chimera software was used to model the 3D structure of RBD (Eswar et al., 2008) (Pettersen et al., 2021).

### *In silico* mutagenesis and variants superimposition

The newly emerged XBB sub-variant of omicron has a total of 14 mutations in the spike protein, including 9 mutations in RBD (G339H, R346T L368I, V445P, G446S, N460K, F486S, F490S, R493Q) and 5 mutations (Del144, V83A, H146Q, Q183E, V213E) in the NTD region (Wang et al., 2022b). According to the literature, spike protein plays a crucial structural and functional role in human pathophysiology (Suleman et al., 2021). Here, we used an *in silico* mutagenesis technique to simulate the structural variations and understand their impact on binding and pathogenesis. To analyze the effect of the aforementioned mutations on the structure and function of NTD and RBD proteins, we used the chimera software to model these mutations in the wild-type structures. Finally, to determine the structural differences between wild-type and mutant NTD and RBD, we superimposed the mutant proteins on the wild-type and recorded the differences as Root Mean Square Deviation (RMSD).

### Bonding network analysis by molecular docking approach

The HADDOCK (high ambiguity driven protein-protein docking) (https://wenmr.science.uu.nl/haddock2.4/) server was used to analyze the effect of identified mutations on the bonding network between wild-type and XBB RBD with human ACE2 receptor. To perform the docking process, the HADDOCK server uses the ambiguous interaction restraints (AIRs) from the expected protein interfaces, and this property distingue HADDOCK from the other *ab initio* docking approaches. The HADDOCK server executes the docking of protein-DNA/RNA, protein-protein, and protein-ligand (Dominguez et al., 2003). In the present study, we performed the restraint docking between RBD and ACE2 by defining the interaction residues 449:A, 453:A, 455:A, 456:A, 486:A, 487:A, 489:A, 493:A, 496:A, 498:A, 500:A, 501:A, 502:A, 505:A for RBD and 21:B, 24:B, 27:B, 28:B, 30:B, 35:B, 38:B, 79:B, 80:B, 82:B, 83:B, 353:B for ACE2 (A and B represents the chain name) [41]. Afterward, the HADDOCK-generated complexes were submitted to the PDBsum web server (http://www.ebi.ac.uk/thornton-srv/databases/pdbsum/Generate.html) (Laskowski and Thornton, 2022) to visualize the bonding interface such as hydrogen bonds, salt bridges, and non-bonded contacts. As the sub-variant XBB has been reported to be involved in immune evasion by escaping the human neutralizing antibodies therefore to verify this statement, we also performed the docking of wild-type and mutant NTD with human mAb. Restraint docking was performed by defining the interface residues 25-32:A, 51-58:A, 100-116:A for mAb, and 145-150:B for NTD (Khan et al., 2022). Similarly, the generated complexes were submitted to the PDBsum server to calculate the hydrogen bonds, salt bridges, and non-bonded contacts.

### Dissociation constant (KD) analysis

The binding strength of a biological complex demonstrates a better understanding of biological activities in a specific pathway, pathogenesis, and therapeutic strategies (Khan et al., 2022). Therefore, to provide more authentic information about the docking complexes (wild-type RBD-ACE2, XBB RBD-ACE2, wild-type NTD-mAb, XBB NTD-mAb), we calculated the KD value by using the online web server PRODIGY (protein binding energy prediction) (https://wenmr.science.uu.nl/prodigy/). The PRODIGY server provides services for calculating binding energies for protein-protein and protein-ligand complexes as well as the identification of binding interfaces (Xue et al., 2016).

### Molecular dynamics of wild-type and XBB variants complexes

To check the structural confirmations of generated complexes in a dynamic environment, we performed the molecular dynamic simulation of wild-type and mutant complexes by using the amber20 package with FF19SB force field (Salomon-Ferrer et al., 2013) (Case et al., 2005). Both the wild-type and mutant complexes were solvated in a Tip3 water box (10Å) and neutralized by adding the Na^+^ and Cl^−^ ions. Furthermore, to remove the bad clashes from the system we performed the two-step gentle minimization. In the first step, we achieved minimization by using the steepest decent and conjugated gradient algorithms for 12,000 and 6,000 cycles, respectively. However, in the second step, the minimization was carried out for 6,000 and 3,000 cycles (Watowich et al., 1988; Meza, 2010). After minimization, the system was equilibrated and heated at 1 atm constant pressure and 300 K, respectively. Finally, the molecular dynamic simulation of 50 ns was performed by using the particle mesh Ewald algorithm for long-range electrostatics interactions and the SHAKE algorithm to treat covalent interaction. Then the trajectories generated by MD simulation were subjected to the post-simulation analysis (Salomon-Ferrer et al., 2013).

### Post-simulation analysis

We used CPPTRAJ and PTRAJ packages to analyze the effect of the XBB variant on the dynamic stability, flexibility, compactness, and hydrogen bonding network of the wild-type and mutant complexes (Roe and Cheatham, 2013). The radius of gyration (Rg) was calculated to analyze the structural compactness during the time of the simulation. The structural dynamic stability was analyzed by calculating the Root Mean Square Deviation (RMSD).

The following mathematical formula was used to calculate the RMSD:

$$RMSD=\sqrt{\frac{\sum_{i=0}^{N}\left[m_{i}*{\left(X_{i}-Y_{i}\right)}^{2}\right]}{M}}$$ (i)

Where N is the number of atoms, m_i_ is the mass of atom *i*, X_i_ is the coordinate vector for target atom *i*, Y_i_ is the coordinate vector for reference atom *i*, and M is the total mass. On the other hand, to analyze the structural flexibility at the residues level, we calculated the Root Mean Square Fluctuation (RMSF). During the time of simulation, the RMSF is a calculation of residual fluctuation instead of positional differences of the entire complex.

### Binding free energy calculation

To calculate the binding free energies of both wild-type and mutant RBD-ACE2 and NTD-mAb complexes in real-time, we used the MM/GBSA approach (Weng et al., 2019). The MM-GBSA approach was previously identified as best for estimations of binding free energies of different biological complexes such as protein-protein, protein-nucleic acid, and protein-ligand. The MMGBSA.py script was used to execute the binding free energy in terms of electrostatic, vdW, SA, and GB of wild-type and mutant complex.

The following mathematical equation was used to calculate free energy:

$$“\Delta G\left(bind\right)=\Delta G\left(complex\right)-\left[\Delta G\left(receptor\right)+\Delta G\left(ligand\right)\right]”$$ (ii)

However, the following equation was used to calculate each component of total free energy separately:

$$\prime \prime G=Gbond+Gele+GvdW+Gpol+Gnpol^{\prime \prime }$$ (iii)

Where the Gbond, Gele, and GvdW represent the bonded, electrostatic, and van der Waals interaction while the Gpol and Gnpol represent the polar and non-polar free energies.

## Results and discussion

### 3-Dimensional structural modeling of XBB variants

**FIGURE 1:**
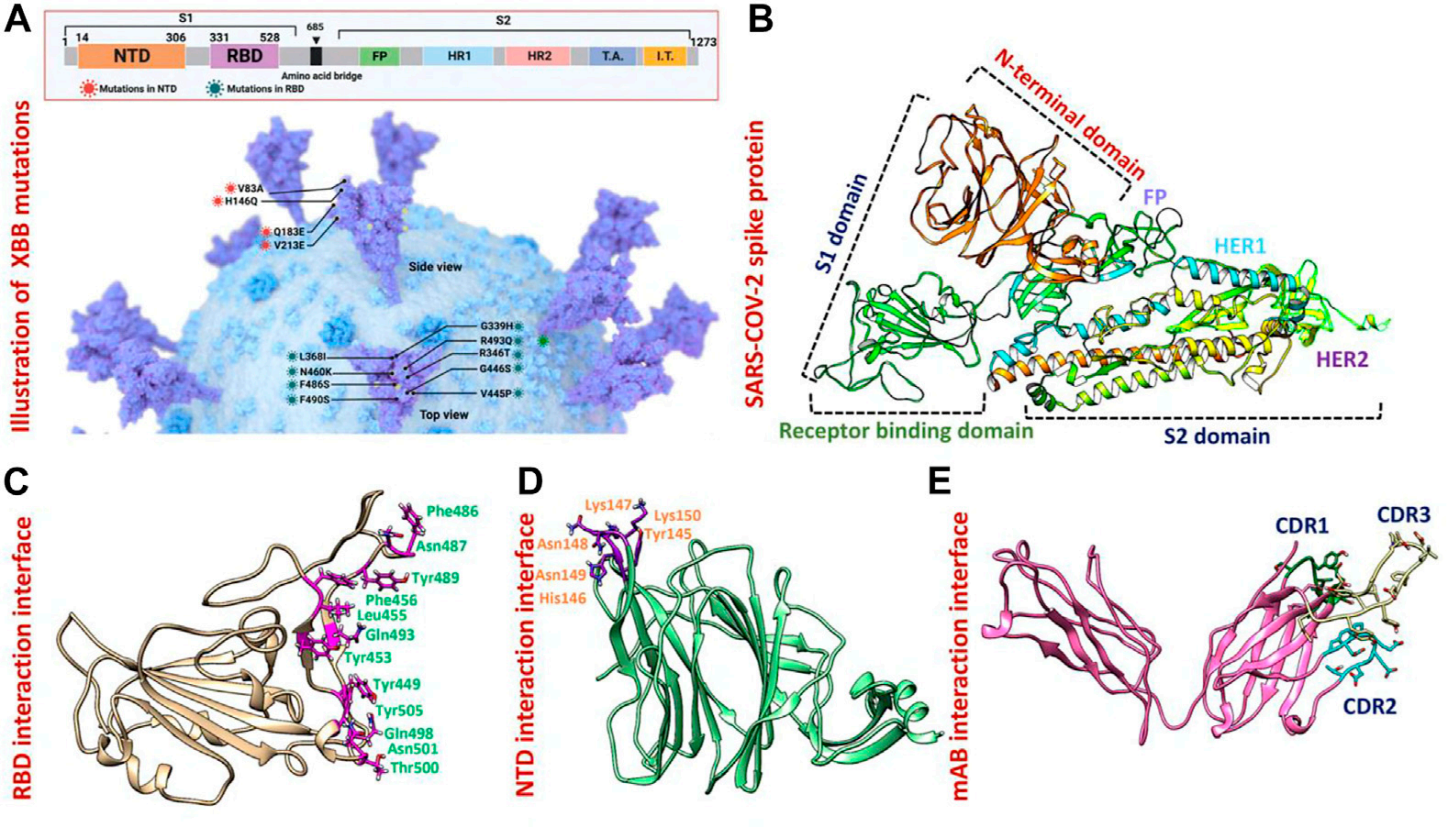
Mutational organization of XBB variant NTD and RBD domains. **(A)** Shows newly reported mutations of XBB in NTD and RBD regions of the spike protein. **(B)** Represents the different domains of SARS-COV-2 spike protein. Different colors represent different domains of the spike protein. **(C)** Shows the interacting amino acid residues of RBD. **(D)** Shows the interacting amino acid residues of NTD. **(E)** Shows the different CDRs regions of human-neutralizing antibodies.

The appearance of the Omicron variant and its further sub-variants continues to rage and contribute to the ongoing coronavirus disease 2019 (COVID-19) pandemic (Lihong and Nair Manoj, 2021) (Wang et al., 2022c) (Mutesa et al., 2021) (Wang et al., 2022d) (Wang et al., 2022e). Numerous sub-linages of the Omicron variant have appeared and are contending in the so-called “variant soup,” despite the fact that the BA.5 sub-variant is currently dominating globally (Lin et al., 2022). It is now clear that BA.5 is being swiftly displaced by four other sub-variants, suggesting the possibility of even another wave of infections in the months to come. Previously reported strains of SARS-CoV-2 in South Africa, United Kingdom and Brazil are 70% more contagious and infectious than the Wuhan strain. As shown in Figure 1A spike glycoprotein has different regions specified for different functions such as the receptor binding domain (RBD) responsible for binding with the human ACE2 receptor which enhances the fusion of viral membrane and host cell. Another important region of spike protein is NTD (N-terminal domain) responsible for binding with the human neutralizing antibodies and has a critical role in the regulation pathogenesis (Huang et al., 2020) (Figure 1B). The previously reported strain B.1.618 has deletions in NTD at the position of Tyr145 and His146, while, point mutations E484K and D614G in the RBD and furin binding site, respectively. These mutations were reported to alter the binding efficiency of RBD and reduced the affinity of NTD with human antibodies (Khan et al., 2021a). The evolution of these variants indicates that the strain that first appeared in Wuhan has subjected to more genetic pressure that has pushed the virus to acquire mutations that may lead to altered infectivity, transmission, and treatment approaches. The newly emerged XBB sub-variant of SARS-CoV-2 was first reported in August in India and swiftly took over in Singapore, India, and other parts of Asia. The XBB variant was found to be more contagious and has immune evasion characteristics due to novel mutations in the N-terminal domain of spike protein. Concerns have been raised that the quick emergence of these sub-variants and their wide variety of spike mutations may further jeopardize the effectiveness of existing COVID-19 vaccines and monoclonal antibody therapies (Wang et al., 2022b). Therefore, it is essential to investigate the atomic level features responsible for higher binding with the host or evasion of the immune response. Hence, the current study provides atomistic insights into the XBB 1.5 variant interaction with the host receptor and antibodies binding using molecular modeling and simulation approaches. Due to the indispensable role of RBD and NTD of spike protein in the regulation of SARS-CoV-2 pathogenesis and immune evasion, we analyzed the binding efficiencies of both wild-type and mutant RBD and NTD with the human ACE2 and mAb, respectively. We retrieved the wild-type complex of the N-terminal domain (NTD) and human monoclonal antibody (4A8) from the UniProt database (Figures 1D, E) and the sequence of RBD (319–541aa) from the wild-type spike protein (P0DTC2) available in the UniProt database (Magrane, 2011). Then the modeler 14.0 embedded in chimera software was used to model the 3D structure of RBD (Eswar et al., 2008) (Pettersen et al., 2021) (Figure 1C).

### Structural analysis of the wild type and XBB variant

**FIGURE 2:**
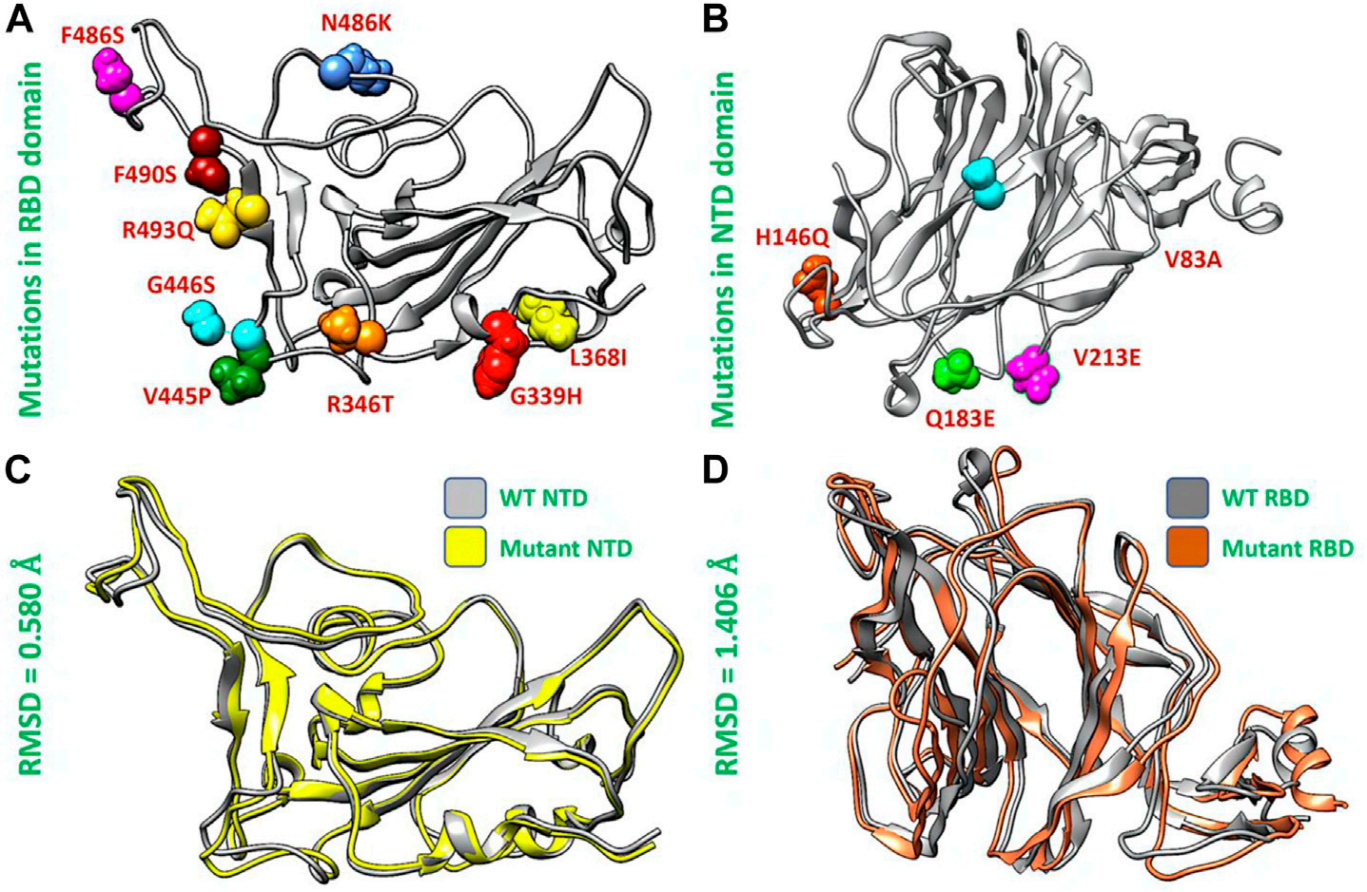
Mutants modeling and superimposition of wild type and mutant NTD and RBD proteins. **(A)** Represents the modeled mutations in RBD by different colors. **(B)** Represents the modeled mutations in NTD by different colors. **(C)** Shows the superimposed wild type and mutant RBD structure. **(D)** Shows the superimposed wild type and mutant NTD structure.

Afterward, we used the chimera software to model the newly emerged mutations G339H, R346T L3b68I, V445P, G446S, N460K, F486S, F490S, R493Q) in RBD (Figure 2A) and Del144, V83A, H146Q, Q183E, V213E mutations in the NTD region of spike protein (Figure 2B). To investigate the impact of these mutations on the structural deviation, we superimposed the mutant proteins on the wild-type protein and the differences in the RMSD were recorded. The wild-type NTD and XBB NTD exhibited little deviation with an RMSD difference of only 0.580 Å (Figure 2C) however, the extensive structural deviation was found in the wild-type RBD and XBB RBD with an RMSD of 1.406 Å (Figure 2D). As the modeled mutations have a significant effect on the structure of both RBD and NTD which may affect the binding of human ACE2 and mAb. To further investigate the effect of these mutations on the bonding network of RBD-ACE2 and NTD-mAb, we performed the molecular docking analysis.

### RBD-ACE2 bonding network analysis

**FIGURE 3:**
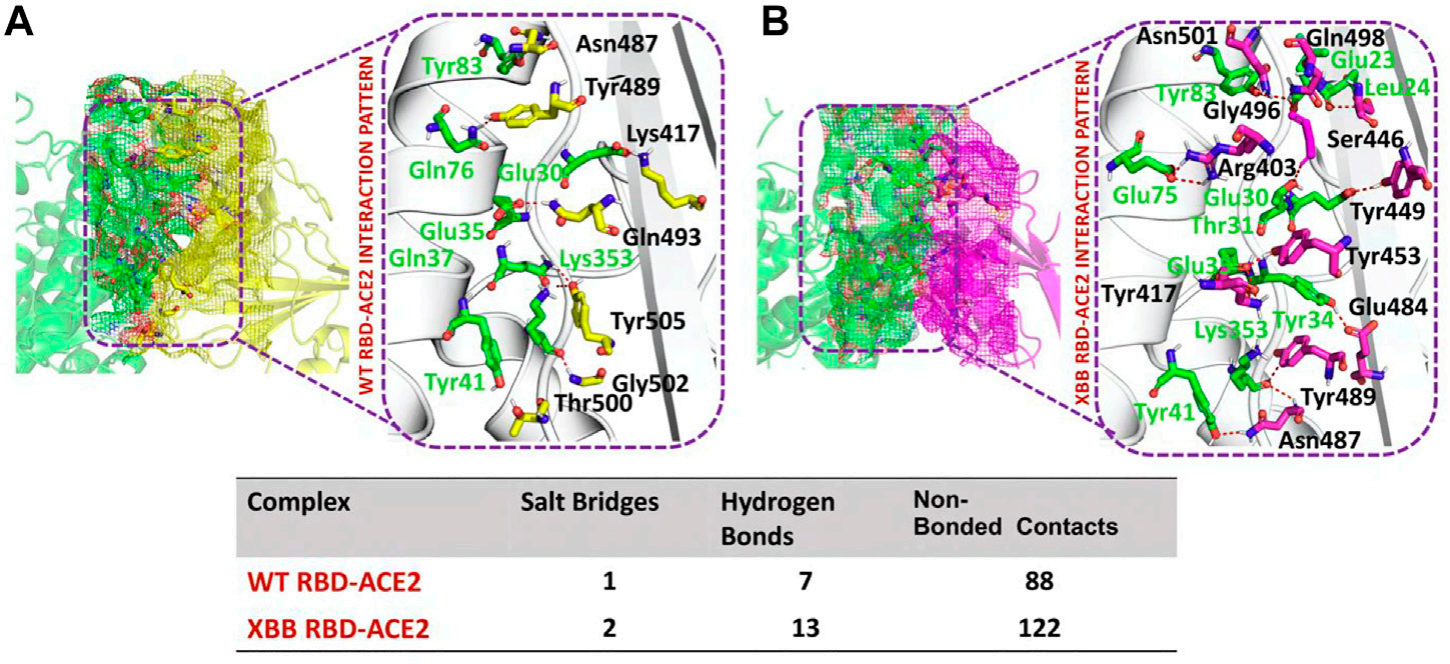
Comparative bonding network analysis of wild-type and mutant RBD-ACE2 complexes. **(A)** Sticks representation of hydrogen bonds in wild-type RBD-ACE2 complex. The green color represents ACE2 and the yellow color represents RBD **(B)** sticks representation of hydrogen bonds in mutant RBD-ACE2 complex. The green color represents ACE2 and the magenta color represents RBD.

To identify the fundamental mechanism behind the higher infectivity and immune evasion of omicron sub-variant XBB, we performed the comparative binding of wild-type and mutant (XBB variants) spike protein by using the HADDOCK server (Table 1). The HADDOCK predicted score for the wild-type RBD-ACE2 complex was −115.0 ± 1.5 kcal/mol. The binding interface analysis of the wild-type RBD-ACE2 complex by PDBsum sever revealed 7 hydrogen bonds, 1 salt bridge, and 88 non-bonded contacts. The key amino acid residues involved in the hydrogen bonds formation include Tyr83-Asn487, Tyr41-Thr500, Lys353-Gly502, Gln37-Tyr505, Glu30-Lys417, and Gln76-Tyr489, while the salt bridge formed between Glu30 and Lys417 residues (Figure 3A). We also docked the mutant (XBB) RBD and ACE2 to compare the binding network with the wild-type RBD-ACE2 complex. The binding score predicted by the HADDOCK server for the mutant RBD-ACE2 complex was −120.8 ± 3.4 kcal/mol. This shows a comparatively slight increase in the docking scores as the Omicron parent and other sub-variants have reported a tighter binding than XBB and wild-type (Wang et al., 2022f; Khan et al., 2022). The interface analysis by PDBsum revealed that the XBB mutation significantly enhanced the binding affinity of RBD with the human ACE2 receptor by making 2 salt bridges, 13 hydrogen bonds, and 122 non-bonded contacts. The hydrogen bonds formed between Tyr41-Asn487, Lys353-Tyr489, Tyr34-Glu484, Glu35-Lys417, Glu35-Tyr453, Thr31-Gly496, Glu30-Tyr449, Glu75-Arg403, Leu24-Gln498, Tyr83-Gln498, Tyr83-Asn501 and Glu23-Ser446 amino acid residues while the salt bridges were formed between Glu35-Lys417 and Glu75-Arg403 residues (Figure 3B). For instance, the Glu35-Lys417, Glu30-Tyr449, Lys353-Tyr489, and Tyr83-Asn501 are strongly conserved with other variants and the wild type (Khan et al., 2021b; Khan et al., 2021c). Interestingly, Glu75-Arg403 which is involved in both hydrogen bond and salt-bridge interaction was observed to be unique to the XBB variant only but not others. Moreover, significant differences in the electrostatic and van der Waals energies of the wild-type and XBB RBD complexes were noted. In particular, the wild-type RBD-ACE2 complex had an electrostatic energy of −184.6 ± 15.2 kcal/mol, while the XBB mutant had a higher electrostatic energy of −218.2 ± 31.4 kcal/mol. Surprisingly this notion was also recorded in the previous variants where electrostatic energy fluctuation was the prime factor in the enhanced binding of RBD with the host receptor, i.e., ACE2 (Khan et al., 2021b; Khan et al., 2021c; Khan et al., 2022). Similarly, van der Waals energies for wild-type and mutant RBD-ACE2 complex were −54.1 ± 2.4 kcal/mol and −65.5 ± 7.3 kcal/mol which shows the higher van der Waals in the case of mutant RBD-ACE2 complex (Table 1). These findings suggest that the stronger binding of the mutant complexes is mainly due to electrostatic and van der Waals interactions and that the differences in interaction conformation may contribute to higher infectivity.

**TABLE 1:** Molecular docking and dissociation constant (K_D_) analysis of the wild type (RBD and NTD) and XBB variant (RBD and NTD) using HADDOCK and Prodigy servers.

Parameters	Wild type NTD	XBB NTD	Wild type RBD	XBB RBD
HADDOCK score	−113.2 ± 0.7	−76.2 ± 2.3	−115.0 ± 1.5	−120.8 ± 3.4
Cluster size	178	87	43	85
RMSD (Å)	0.4 ± 0.3	12.5 ± 0.7	14.6 ± 0.3	0.9 ± 0.6
Van der Waals energy	−58.2 ± 2.5	−34.9 ± 4.0	−54.1 ± 2.4	−65.5 ± 7.3
Electrostatic energy	−246.3 ± 4.0	−182.4 ± 12.9	−184.6 ± 15.2	−218.2 ± 31.4
De-solvation energy	−13.1 ± 1.8	−14.4 ± 0.8	−25.2 ± 3.0	−13.3 ± 1.7
Restraint’s violation energy	74.6 ± 18.4	95.7 ± 28.5	12.1 ± 16.4	16.0 ± 22.2
Buried surface area (A2)	1,529.7 ± 19.2	1,212.8 ± 78.2	1837.7 ± 64.1	1898.5 ± 101.7
Z-score	−1.4	−0.3	−1.3	−1.9
KD (dissociation constant)	6.1E^−09^	1.9E^−08^	2.1E^−8^	18.1E^−10^

### NTD-mAb bonding network analysis

**FIGURE 4:**
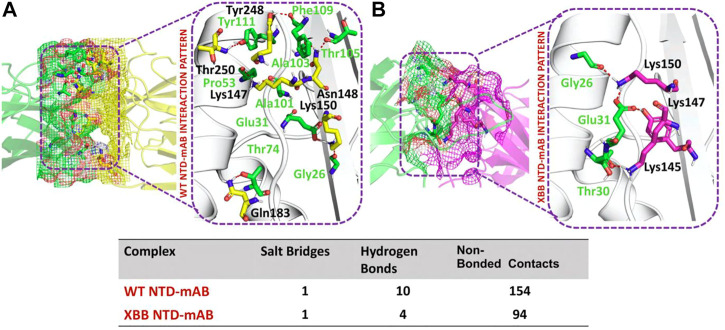
Comparative bonding network analysis of wild-type and mutant NTD-mAb complexes. **(A)** Sticks representation of hydrogen bonds in wild-type NTD-mAb complex. The green color represents antibody and the yellow color represents NTD **(B)** sticks representation of hydrogen bonds in mutant NTD-mAb complex. The green color represents antibody and the magenta color represents NTD.

According to a recent study, the neutralization of XBB by sera from individuals who had been vaccinated or infected was significantly impaired. This included sera from individuals who had received a booster shot with the WA1/BA.5 bivalent mRNA vaccine. The titers against XBB sub-variants were 66–155 times lower than previously observed. Monoclonal antibodies that were effective at neutralizing the original Omicron variant were largely ineffective against the new sub-variants. These findings suggest that there may be a reduced immune response to the new XBB sub-variants (reference). Therefore, to check the effect of the XBB variant on the bonding network between NTD and neutralizing antibodies, we performed the docking of wild-type and XBB-NTD with human mAb. The binding score predicted by HADDOCK for the wild-type NTD-mAb complex was −113.2 ± 0.7 kcal (Table 1), while the interface analysis by PDBsum revealed 1 salt bridge, 10 hydrogen bonds, and 154 non-bonded contacts between the generated complex. Among the hydrogen bonds, Tyr111-Thr250, Pro53-Lys147, Ala101-Lys147, Phe109-Tyr248, Thr105-Asn148, Ala103-Asn148, Glu31-Lys150, Gly26-Lys150 and Thr74-Gln183 amino acid residues were involved, while the salt bridge was formed between Glu31-Lys150 residues (Figure 4A). A similar finding has been previously observed by a study investigating B.1.618 variant {Khan, 2021 #135}. The binding pattern of XBB NTD with the mAb was also evaluated to check the effect of detected mutations on the binding efficiency of NTD with mAb. Significant differences were observed in the binding network of XBB NTD with mAb as compared to the wild-type complex. The binding score of mutant NTD-mAb complex generated by HADDOCK was −76.2 ± 2.3 kcal/mol with only a single salt bridge, four hydrogen bonds and 94 non-bonded contacts were observed by analyzing the binding interface between NTD and mAb. The amino acid residues involved in the hydrogen bonds formation were Thr30-Lys147, Glu31-Tyr145, Glu31-Lys150, and Gly26-Lys150, while the residues Glu31-Lys150 were involved in the salt bridge formation (Figure 4B). Although these interactions target the essential residues previously described for neutralization but the weaker binding of mAb due to loss of many contacts may release the mAb soon as a mechanism of evasion by the spike protein thus point essential features for future therapeutics development. We also explored the variations in terms of electrostatic and vdW energies. The wild-type NTD-mAb complex had an electrostatic energy of −246.3 ± 4.0, while the mutant had a lower electrostatic energy of −182.4 ± 12.9 kcal/mol. Similarly, a vdW for the wild-type and XBB RBD-NTD complex were −58.2 ± 2.5 kcal/mol and −34.9 ± 4.0 kcal/mol, respectively, which shows a significant decrease in the van der Waals energy in the case of mutant mAb-NTD complex (Table 1). The above data shows that the immune evasion properties of the XBB variant may be due to the weak electrostatic and van der Waals interactions and that the differences in the hydrogen bonding network may contribute to higher infectivity.

### Molecular dynamics simulation (MDS) of the complexes

#### Dynamics of the wild-type and XBB NTD complexes with mAb

**FIGURE 5:**
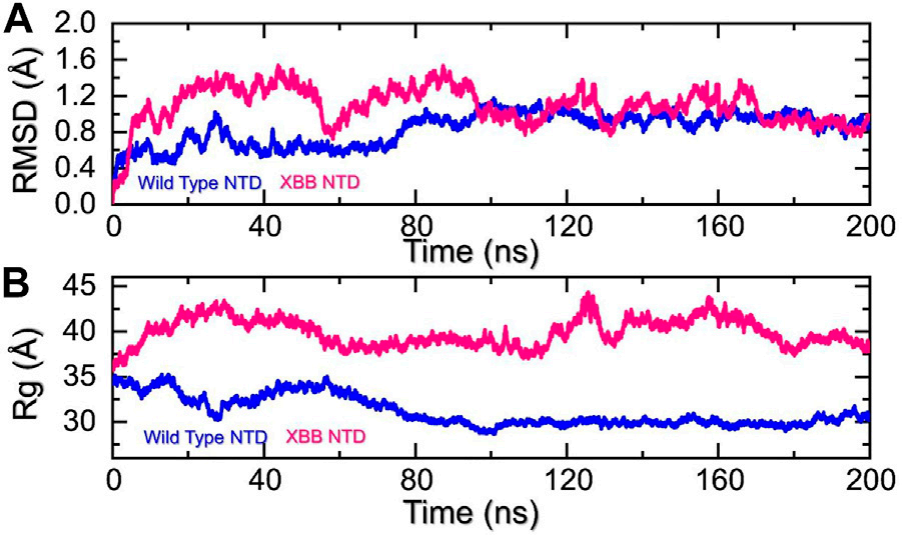
Stability and compactness analysis by RMSD and Rg. **(A)** represent the RMSD values of wild-type and XBB NTD-mAB complex. **(B)** Represent the Rg values of wild-type and XBB NTD-mAb complex.

Molecular dynamics (MD) simulation is an important approach for studying molecular mechanisms because it allows researchers to observe the dynamics of the complex in atomic detail. This can provide insights into the mechanisms of protein-protein interactions, such as how the proteins bind to each other, how they change conformation, and how they transfer information. MD simulation can also be used to predict the structures of protein-protein complexes that are difficult or impossible to study experimentally, such as complexes that are transient or unstable. Additionally, MD simulation can be used to design new drugs that target protein-protein interactions by identifying potential binding sites and understanding the interactions between the drug and the target proteins. Recent studies have highlighted the importance of MD simulation in understanding the molecular mechanisms of protein-protein interactions in COVID-19 (ref). In the present study, we performed the molecular dynamics simulation to analyze the stability, residual flexibility, compactness, and average hydrogens bonds of RBD-ACE2 and NTD-mAb complexes. RMSD (root mean square deviation) is a measure used to quantify the difference between the initial and final conformations of a protein’s backbone. It is often used in molecular dynamics simulations to assess the stability of a protein over time. A low RMSD value indicates a more stable structure, while a high RMSD value suggests greater deviation and less stability. In this context, the RMSD of the Cα backbone was calculated for a 200 ns trajectory of each protein-protein complex to estimate the stability of the complex over time. As Figure 5A shows, both the wild type-NTD-mAb and XBB-mAb complexes exhibited a stable dynamic. We found that the wild-type NTD-mAb complex had a stable dynamic throughout the simulation, reaching equilibrium at 80 ns and maintaining stability at 1 Å. However, the XBB NTD-mAb complex had higher deviations between 10 and 80 ns, indicating a less stable dynamic. The stable dynamic behavior of the wild-type NTD-mAb complex throughout the simulation time indicates that substitution in the NTD of the XBB variant decreased the structural stability of the NTD-mAb complex that allows it to evade binding with human neutralizing antibodies. Our findings are consistent with the previous study, which indicated that the XBB variant has immune evasion properties (Wang et al., 2022b). Moreover, the destabilization of NTD and its association with the loss of essential contacts and immune evasion has been previously recorded in other variants too (ref). Our study also analyzed structural compactness in a dynamic environment by calculating the radius of gyration (Rg) as a function of time. The results, shown in Figure 5B, indicate that the wild-type NTD-mAb complex had a uniform Rg with a mean Rg of 30 Å, while the XBB NTD-mAb complex had a higher Rg value of 40.0 Å and experienced significant structural changes. It can be seen that the wild-type and XBB variant had significant variations in the compactness. This shows that loss of essential contacts may help to release the mAb from the NTD and causing increase in the protein size during the simulation. This suggests that the XBB NTD-mAb complex underwent significant binding and unbinding events during the simulation. This implies that due to the acquired mutations the NTD has endured structural rearrangements which consequently causes dynamic variations and resistance to mAb.

**FIGURE 6:**
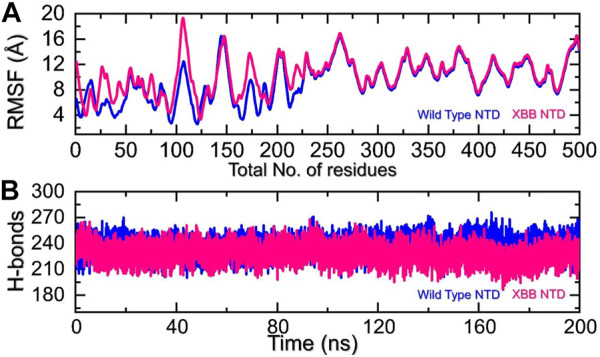
Residual fluctuation and hydrogen bonding analysis. **(A)** Represent the RMSF values of wild-type and XBB NTD-mAb complex. **(B)** Represent the average hydrogen bonds of wild-type and XBB NTD-mAb complex.

We analyzed the fluctuations of the wild type and variants at the residue level by looking at local-level flexibility. This can affect intermolecular binding and molecular recognition, and potentially impact the overall function of the biological molecule. A higher RMSF value indicates a more flexible region, while a lower value indicates a more stable region. Loops tend to be more unstable due to their lack of fixed secondary structure, resulting in a higher RMSF. In comparison to the wild-type NTD-mAb, the XBB NTD-mAb showed significant variations in residual flexibility between amino acids 1-225 (Figure 6A). The variations in conformational optimization and binding strength were also demonstrated by the differential flexibility index. To evaluate the changes in hydrogen bonding interactions during a simulation, the total number of hydrogen bonds between interacting molecules can be calculated at different time points. This information can be used to determine the binding strength of the molecules by observing how the number of hydrogen bonds changes over the course of the simulation. For instance, hydrogen bonding is a key estimator in determining the association of molecular networks joined by proteins through such contacts. Thus, analyzing the hydrogen bonding patterns can provide insights into how the molecules interact and how these interactions change over time. Figure 6B illustrates that the average number of hydrogen bonds in the XBB NTD-mAb complex is lower than that of the wild-type NTD-mAb complex, as observed over the course of the simulation. In particular average number of hydrogen bonds in the wild-type NTD-mAb complex was calculated to be 248 while in the XBB-NTD-mAb complex average number of hydrogen bonds was calculated to be 242 which shows a significant reduction in the average number of hydrogen bonds in the XBB variant. This supports the conclusions drawn from the molecular docking results, which predicted that the XBB NTD-mAb complex had weaker binding interactions than the wild-type NTD-mAb complex. This further demonstrates that the simulation results are consistent with the predictions made by the molecular docking study.

### Dynamics of the wild-type and XBB RBDD complexes with hACE2

**FIGURE 7:**
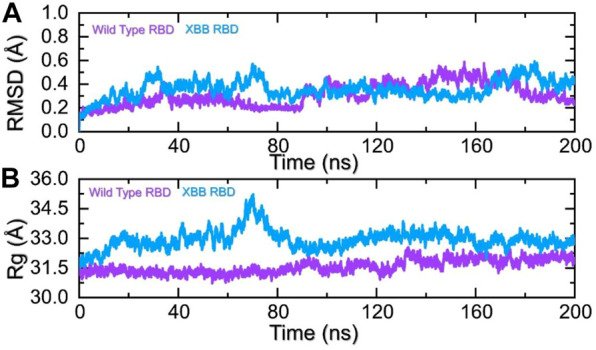
Stability and compactness analysis by RMSD and Rg. **(A)** represent the RMSD values of wild-type and XBB RBD-ACE2 complex. **(B)** Represent the Rg values of wild-type and RBD-ACE2 complex.

To identify the fundamental mechanism behind the higher infectivity and immune evasion of omicron sub-variant XBB, we performed the MD simulation to check the dynamic features of wild-type and XBB RBD with the human ACE2 receptor. To check the stability of wild-type and XBB RBD-ACE2 complex, we calculated the RMSD value. As Figure 7A shows, both wild-type RBD-ACE2 and XBB-ACE2 complexes exhibited a stable dynamic. We found that the RMSD value of the XBB-ACE2 complex is higher between 20 and 85 ns as compared to the wild-type, become stable between 90 and 170 ns. The RMSD the value of XBB variant falls in the normal range and verify the stronger affinity towards the human ACE2 receptor. Both the complexes converged with each other soon after reaching 20 ns which shows that both the structures have attained the similar atomic configuration thus showing the dynamic accuracy of the approach. Moreover, our findings are consistent with the previous study, which indicated that the XBB variant has the stronger binding affinity with the human ACE2 receptor (Wang et al., 2022b). Furthermore, the compactness of the wild-type and XBB variant followed a similar pattern however a little perturbation was observed in the XBB-ACE2 complex 50–80 ns of simulation (Figure 7B) during the 1–100 ns the Rg difference between the wild type and XBB remained higher however the Rg of the XBB variant decreased and the protein size decreased with time. From 101 to 200 ns the Rg of XBB remained more comparable with the wild type with no significant perturbation in the Rg. These results of RMSD and Rg strongly align with each other thus showing the accuracy of the approach too. The variations in the protein size/compactness justify the binding affinity and infectivity of XBB in contrast to the wild type.

**FIGURE 8:**
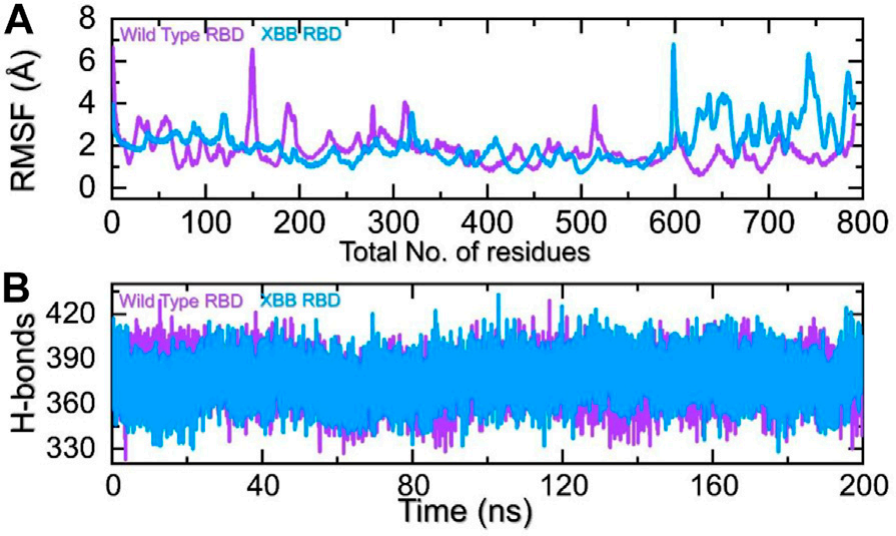
Residual fluctuation and hydrogen bonding analysis. **(A)** Represent the RMSF values of wild-type and XBB RBD-ACE2 complex. **(B)** Represent the average hydrogen bonds of wild-type and XBB RBD-ACE2 complex.

The comparison of the residual fluctuation between the wild-type and XBB-ACE2 complexes supports our molecular docking findings. Figure 8A illustrates that while the wild-type RBD-ACE2 complex experiences more fluctuation in the 100-300aa range, it is less pronounced in the XBB variant. On the other hand, both complexes demonstrate increased fluctuation in the 600-800aa region, which may be caused by the high RMSF of the terminal amino acids. In particular, the region 600–796 is RBD which shows higher fluctuation in the XBB variant in contrast to the wild type. The higher fluctuation in the XBB variant shows structural optimization for binding which is consistent with the previous studies. This region exhibits the loops, i.e., 474-505 (746–776) are responsible for interaction with ACE2 demonstrated higher fluctuation which corroborate with previous findings. These observations suggest that the RBD protein has undertaken significant structural changes, which enhance its binding capabilities and augment its infectivity. We also calculated the total number of hydrogen bonds between interacting molecules at different time points for both wild-type and XBB RBD-ACE2 complexes. As Figure 8B shows that the average hydrogen bonds of XBB RBD-ACE2 complex is higher as compared to the wild-type RBD-ACE2 complexes which further verified the previous study and our molecular docking data. In the case of wild-type RBD in association with ACE2 an average number of hydrogen bonds were calculated to be 387 while in XBB-RBD coupled with ACE2 demonstrated 392 average hydrogen bonds. In the wild type significant reduction in the hydrogen bonds after 80 ns. These variations in the hydrogen bonding network further validate the docking results which shows better affinity of XBB-RBD for host ACE2 than the wild type.

### Binding free energy estimation through MM/GBSA

The traditional alchemical and other methods suffer from accuracy, resources requirements and time consumption. The contemporary MM/GBSA approach overcome such limitations with lower cost and computational resources. It has been used in wide range of studies to estimate the binding free energy of different biological complexes. For instance, calculation of binding free energy for RBD and ACE2 from various variants, i.e., B.1.1.7, B.1.617, B.1.618, and B.1.529 accurately determined interaction affinity and mechanism. Therefore, the current study also uses MM/GBSA approach to estimate the binding free energy for RBD and NTD (wild type and XBB variant) using the 200 ns simulation trajectory. As given in Table 2, the vdW for the wild type-RBD complex with ACE2 was estimated to be −95.57 kcal/mol while for XBB-RBD in complex with ACE2 demonstrated a vdW of −114.30 kcal/mol. This shows significant increase in the vdW in the XBB variant in contrast to the wild type. The increased vdW is a notion from previous studies based on different variants of SARS-CoV-2. Moreover, the electrostatic energy is the key factor previously reported to be responsible for the enhanced binding of different variants. In the current study similar observations were seen as the wild type reported an electrostatic energy of −582.70 kcal/mol while the XBB-RBD variant reported an electrostatic energy of −640.42 kcal/mol which align with the previous findings. The total binding energy for the wild-type RBD in complex with ACE2 reported −50.10 kcal/mol while XBB-RBD coupled with ACE2 reported −52.66 kcal/mol, respectively. This shows though the binding of XBB is slightly increased but due to the variation in the bonding network and other factors makes the XBB variant to enter into the host cell efficiently than the wild type. On the other hand, the NTD-mAb complexes demonstrated opposite results than the RBD-ACE2. The vdW for wild type NTD-mAb was calculated to be −93.66 kcal/mol while for XBB NTD-mAb reported a vdW of −66.26 kcal/mol. Moreover, the electrostatic differences were calculated to be −811.17 kcal/mol for the wild type while −482.37 kcal/mol for XBB NTD-mAb complex was reported. The total binding free energy for the wildtype NTD-mAb was calculated to be −65.94 kcal/mol while for XBB NTD-mAb was reported to be −35.06 kcal/mol, respectively. The significant difference in the total binding energy factors explains that the XBB variant possess stronger immune evasion properties than the others variants and wild type. The current findings also align with the experimental results which reports immune evasion features of XBB variant. Thus, this study accurately explored the atomic features for host interaction and immune evasion. The binding free energy results for each complex is shown in Table 2.

**TABLE 2:** Binding free energy results estimated as MM/GBSA. All the energies are given in kcal/mol and kj/mol.

Parameters	Wildtype RBD		XBB RBD		Wildtype NTD		XBB-NTD	
vdW	−95.57	−399.86	−114.30	−478.23	−93.66	−391.87	−66.26	−277.23
Electrostatic	−582.70	−2,438.02	−640.42	−2,679.52	−811.17	−3,393.94	−482.37	−2018.24
GB	640.53	2,679.98	717.46	3,001.85	850.35	3,557.86	522.42	2,185.81
SA	−12.36	−51.71	−15.40	−64.43	−11.45	−47.91	−8.85	−37.03
ΔG total	−50.10	−209.62	−52.66	−220.33	−65.94	−275.89	−35.06	−146.69

## Conclusion

The current XBB variant of SARS-CoV-2 with the strongest immune escaping properties is currently the most dominant variant circulating around the world. In the current scenario, it was highly required to delineate the binding capabilities of NTD of XBB subvariant towards human neutralizing antibodies and to dig out the binding affinity of RBD of XBB subvariant with ACE2 receptor. The current study uses molecular interaction and simulation-based approaches to decipher the binding mechanism of RBD with ACE2 and mAb interaction with NTD of the spike protein. Molecular docking of the wild type NTD with mAb, XBB NTD docking with mAb, wild type RBD docking with ACE2 and XBB RBD with ACE2 receptor demonstrated variation in the interaction network, i.e., hydrogen bonds, salt-bridges and non-bonded contacts. Molecular simulation analysis such as RMSD, RMSF, Rg and hydrogen bonding analysis revealed variation in the dynamics features of the RBD and NTD complexes due to the acquired mutations. Furthermore, the total binding energy revealed that the XBB variant possess stronger immune evasion properties than the others variants and wild type. The current study provides structural features for the XBB variant binding and immune evasion which can be used to design novel therapeutics.

## Data Availability

The original contributions presented in the study are included in the article/Supplementary Material, further inquiries can be directed to the corresponding author.

## References

[B1] Arora P. Zhang L. Krüger N. Rocha C. Sidarovich A. Schulz S. (2022). SARS-CoV-2 Omicron sublineages show comparable cell entry but differential neutralization by therapeutic antibodies. Cell Host Microbe 30, 1130. 10.1016/j.chom.2022.04.01 PMC9072809 35588741

[B2] Callaway E. Ledford H. (2021). How bad is omicron? What scientists know so far. Nature 600 (7888), 197–199. 10.1038/d41586-021-03614-z 34857948

[B3] Callaway H. M. O. V. P. (2022). Scientists on alert. Berlin, Germany: Springer Nature.

[B4] Cao Y. Wang J. Jian F. Xiao T. Song W. Yisimayi A. (2022). Omicron escapes the majority of existing SARS-CoV-2 neutralizing antibodies. Nature 602 (7898), 657–663. 10.1038/s41586-021-04385-3 35016194 PMC8866119

[B5] Carreño J. M. Alshammary H. Tcheou J. Singh G. Raskin A. J. Kawabata H. (2022). Activity of convalescent and vaccine serum against SARS-CoV-2 Omicron. Nature 602 (7898), 682–688. 10.1038/s41586-022-04399-5 35016197

[B6] Case D. A. Cheatham T. E. Darden T. Gohlke H. Luo R. Merz K. M. (2005). The Amber biomolecular simulation programs. J. Comput. Chem. 26 (16), 1668–1688. 10.1002/jcc.20290 16200636 PMC1989667

[B7] Dominguez C. Boelens R. Bonvin A. M. (2003). Haddock: A protein− protein docking approach based on biochemical or biophysical information. J. Am. Chem. Soc. 125 (7), 1731–1737. 10.1021/ja026939x 12580598

[B8] Eswar N. Eramian D. Webb B. Shen M. Y. Sali A. (2008). Protein structure modeling with MODELLER. Methods Mol. Biol. 426, 145–159. 10.1007/978-1-60327-058-8_8 18542861

[B9] Evans J. P. Zeng C. Qu P. Faraone J. Zheng Y. M. Carlin C. (2022). Neutralization of SARS-CoV-2 omicron sub-lineages BA. 1, BA. 1.1, and BA. 2. Cell host microbe 30, 1093. 10.1016/j.chom.2022.04.014 35526534 PMC9035359

[B10] Hu B. Guo H. Zhou P. Shi Z. L. (2022). Author correction: Characteristics of SARS-CoV-2 and COVID-19. Nat. Rev. Microbiol. 20, 315. 10.1038/s41579-022-00711-2 PMC8864972 35197601

[B11] Huang Y. Yang C. Xu X. F. Xu W. Liu S. W. (2020). Structural and functional properties of SARS-CoV-2 spike protein: Potential antivirus drug development for COVID-19. Acta Pharmacol. Sin. 41 (9), 1141–1149. 10.1038/s41401-020-0485-4 32747721 PMC7396720

[B12] Khan A. Gui J. Ahmad W. Haq I. Shahid M. (2021). The SARS-CoV-2 B. 1.618 variant slightly alters the spike RBD–ACE2 binding affinity and is an antibody escaping variant: A computational structural perspective. RSC Adv. 11 (48), 30132–30147. 10.1039/d1ra04694b 35480256 PMC9040812

[B13] Khan A. Waris H. Rafique M. Suleman M. Mohammad A. Ali S. S. (2022). The Omicron (B 1.1 529) variant of SARS-CoV-2 binds to the hACE2 receptor more strongly and escapes the antibody response: Insights from structural and simulation data. Int. J. Biol. Macromol. 200, 438–448. 10.1016/j.ijbiomac.2022.01.059 35063482 PMC8767976

[B14] Khan A. Wei D. Q. Kousar K. Abubaker J. Ahmad S. Ali J. (2021). Preliminary structural data revealed that the SARS-CoV-2 B. 1.617 variant’s RBD binds to ACE2 receptor stronger than the wild type to enhance the infectivity. ChemBioChem 22, 2641–2649. 10.1002/cbic.202100191 34160124 PMC8426803

[B15] Khan A. Zia T. Suleman M. Khan T. Ali S. S. Abbasi A. A. (2021). Higher infectivity of the SARS-CoV-2 new variants is associated with K417N/T, E484K, and N501Y mutants: An insight from structural data. J. Cell. Physiology 236, 7045–7057. 10.1002/jcp.30367 PMC8251074 33755190

[B16] Khoury D. S. Cromer D. Reynaldi A. Schlub T. E. Wheatley A. K. Juno J. A. (2021). Neutralizing antibody levels are highly predictive of immune protection from symptomatic SARS-CoV-2 infection. Nat. Med. 27 (7), 1205–1211. 10.1038/s41591-021-01377-8 34002089

[B17] Laskowski R. A. Thornton J. M. (2022). PDBsum extras: SARS‐Cov‐2 and AlphaFold models. Protein Sci. 31 (1), 283–289. 10.1002/pro.4238 34779073 PMC8662102

[B18] Lihong L. Nair Manoj S. (2021). Striking antibody evasion manifested by the omicron variant of sars-cov-2. Nature 2021, 1–8. 10.1038/s41586-021-04388-0 35016198

[B19] Lin J. Zhang H. Xi L. Liu F. Liu W. Guo Q. (2022). Life meaning constructed from dignity therapy in traditional Chinese culture: A qualitative analysis of dignity therapy generativity documents. J. Biomol. Struct. Dyn. 2022, 1–8. 10.1017/S1478951522001614 36562276

[B20] Magrane M. (2011). UniProt knowledgebase: A hub of integrated protein data. Database 2011, barr009. 10.1093/database/bar009 PMC3070428 21447597

[B21] Meza J. C. (2010). Steepest descent. Wiley Interdiscip. Rev. Comput. Stat. 2 (6), 719–722. 10.1002/wics.117

[B22] Mutesa L. Ndishimye P. Butera Y. Souopgui J. Uwineza A. Rutayisire R. (2021). A pooled testing strategy for identifying SARS-CoV-2 at low prevalence. Nature 589 (7841), 276–280. 10.1038/s41586-020-2885-5 33086375

[B23] Pettersen E. F. Goddard T. D. Huang C. C. Meng E. C. Couch G. S. Croll T. I. (2021). UCSF ChimeraX: Structure visualization for researchers, educators, and developers. Protein Sci. 30 (1), 70–82. 10.1002/pro.3943 32881101 PMC7737788

[B24] Planas D. Saunders N. Maes P. Guivel-Benhassine F. Planchais C. Buchrieser J. (2022). Considerable escape of SARS-CoV-2 Omicron to antibody neutralization. Nature 602 (7898), 671–675. 10.1038/s41586-021-04389-z 35016199

[B25] Roe D. R. Cheatham T. E. III (2013). PTRAJ and CPPTRAJ: Software for processing and analysis of molecular dynamics trajectory data. J. Chem. theory Comput. 9 (7), 3084–3095. 10.1021/ct400341p 26583988

[B26] Salomon-Ferrer R. Götz A. W. Poole D. Le Grand S. Walker R. C. (2013). Routine microsecond molecular dynamics simulations with AMBER on GPUs. 2. Explicit solvent particle mesh Ewald. J. Chem. theory Comput. 9 (9), 3878–3888. 10.1021/ct400314y 26592383

[B27] Salomon-Ferrer R. Case D. A. Walker R. C. (2013a). An overview of the Amber biomolecular simulation package. Wiley Interdiscip. Rev. Comput. Mol. Sci. 3 (2), 198–210. 10.1002/wcms.1121

[B28] Shrestha L. B. Foster C. Rawlinson W. Tedla N. Bull R. A. (2022). Evolution of the SARS-CoV-2 omicron variants BA. 1 to BA. 5: Implications for immune escape and transmission. Rev. Med. Virology 32 (5), e2381. 10.1002/rmv.2381 35856385 PMC9349777

[B29] Suleman M. Yousafi Q. Ali J. Ali S. S. Hussain Z. Ali S. (2021). Bioinformatics analysis of the differences in the binding profile of the wild-type and mutants of the SARS-CoV-2 spike protein variants with the ACE2 receptor. Comput. Biol. Med. 138, 104936. 10.1016/j.compbiomed.2021.104936 34655895 PMC8501515

[B30] Tegally H. Moir M. Everatt J. Giovanetti M. Scheepers C. Wilkinson E. (2022). Emergence of SARS-CoV-2 omicron lineages BA. 4 and BA. 5 in South Africa. Nat. Med. 28 (9), 1785–1790. 10.1038/s41591-022-01911-2 35760080 PMC9499863

[B31] Viana R. Moyo S. Amoako D. G. Tegally H. Scheepers C. Althaus C. L. (2022). Rapid epidemic expansion of the SARS-CoV-2 Omicron variant in southern Africa. Nature 603 (7902), 679–686. 10.1038/s41586-022-04411-y 35042229 PMC8942855

[B32] Wang J. Fatima Muhammad S. Aman S. Khan A. Munir S. Khan M. (2022). Structural communication fingerprinting and dynamic investigation of RBD-hACE2 complex from BA. 1× AY. 4 recombinant variant (Deltacron) of SARS-CoV-2 to decipher the structural basis for enhanced transmission. J. Biomol. Struct. Dyn. 2022, 1–12. 10.1080/07391102.2022.2123399 36129018

[B33] Wang Q. Guo Y. Iketani S. Nair M. S. Li Z. Mohri H. (2022). Antibody evasion by SARS-CoV-2 Omicron subvariants BA.2.12.1, BA.4 and BA.5. Nature 608, 603. 10.1038/s41586-022-05053-w 35790190 PMC9385487

[B34] Wang Q. Iketani S. Li Z. Liu L. Guo Y. Huang Y. (2022). Alarming antibody evasion properties of rising SARS-CoV-2 BQ and XBB subvariants. Cell 186, 279. 10.1016/j.cell.2022.12.018 36580913 PMC9747694

[B35] Wang Q. Iketani S. Li Z. Guo Y. Yeh A. Y. Liu M. (2022). Antigenic characterization of the SARS-CoV-2 Omicron subvariant BA. 2.75. Cell Host Microbe 30 (11), 1512–1517. 10.1016/j.chom.2022.09.002 36108630 PMC9444898

[B36] Wang Q. Li Z. Ho J. Guo Y. Yeh A. Y. Mohri H. (2022). Resistance of SARS-CoV-2 omicron subvariant BA. 4.6 to antibody neutralisation. Lancet Infect. Dis. 22 (12), 1666–1668. 10.1016/S1473-3099(22)00694-6 36328002 PMC9621396

[B37] Wang X.-J. Yao L. Zhang H. Y. Zhu K. L. Zhao J. Zhan B. D. (2022). Neutralization sensitivity, fusogenicity, and infectivity of Omicron subvariants. Genome Med. 14 (1), 146–213. 10.1186/s13073-022-01151-6 36581867 PMC9798359

[B38] Watowich S. J. Meyer E. S. Hagstrom R. Josephs R. (1988). A stable, rapidly converging conjugate gradient method for energy minimization. J. Comput. Chem. 9 (6), 650–661. 10.1002/jcc.540090611

[B39] Weng G. Wang E. Chen F. Sun H. Wang Z. Hou T. (2019). Assessing the performance of MM/PBSA and MM/GBSA methods. 9. Prediction reliability of binding affinities and binding poses for protein–peptide complexes. Phys. Chem. Chem. Phys. 21 (19), 10135–10145. 10.1039/c9cp01674k 31062799

[B40] Xue L. C. Rodrigues J. P. Kastritis P. L. Bonvin A. M. Vangone A. (2016). Prodigy: A web server for predicting the binding affinity of protein–protein complexes. Bioinformatics 32 (23), 3676–3678. 10.1093/bioinformatics/btw514 27503228

